# Massive Middle Cerebral Artery Ischemic Stroke Associated with Severe Acute Respiratory Distress Syndrome in H1N1 Influenza: A Case Report

**DOI:** 10.31729/jnma.6946

**Published:** 2022-02-28

**Authors:** Suju Bhattarai, Bipin Karki, Shweta Pokhrel, Sachit Regmi, Bibek Shrestha, Pramesh Sunder Shrestha

**Affiliations:** 1 Department of Critical Care Medicine, Om Hospital and Research Center, Chabahil, Kathmandu, Nepal; 2 Department of Anaesthesiology, Maharajgunj Medical Campus, Tribhuvan University Teaching Hospital, Kathmandu, Nepal

**Keywords:** *acute respiratory distress syndrome*, *decompressive craniotomy*, *influenza*, *stroke*

## Abstract

Influenza has a common occurrence during its peak seasons. It usually causes disease of the respiratory tract including severe acute respiratory distress syndrome. However, it may also cause disease and complication of other organ systems. We present a rare complication of influenza in which a patient secondary to influenza developed massive middle cerebral artery ischemic stroke. The patient however survived following recovery of both severe acute respiratory distress syndrome and ischemic stroke after decompressive craniectomy and a prolonged intensive care unit stay. This case report is to highlight the importance of influenza related complications besides the pulmonary infliction which can lead to morbidity and even mortality if not managed on time.

## INTRODUCTION

Every year, influenza like illness occurs as epidemic all over the world including Nepal.^1^ Although respiratory symptoms are most commonly associated with influenza, it may also manifest with other non-respiratory complications such as myocarditis, myositis, acute kidney injury (AKI), myocardial infarction (MI), disseminated intravascular coagulation (DIC) and rarely stroke.^2^ We present a case of 46 years male with polymerase chain reaction (PCR) proven H1N1 associated severe acute respiratory distress syndrome (ARDS) who developed massive ischemic stroke during the course of illness.

## CASE REPORT

A 46 year otherwise healthy male presented to the emergency department (ED) with complaints of dry cough and fever for a week with shortness of breath for one day. Fever was acute in onset with a maximum recorded temperature of 103 degrees Fahrenheit.

On examination at the ED, he was restless and cyanosed on arrival. He was tachypneic with a respiratory rate of 50 breaths/min. His arterial oxygen saturation (SpO2) was 70% in ambient air. His blood pressure was 150/104mm of Hg and pulse rate was 120 beats/min. Examination of the respiratory system revealed bilaterally decreased air entry with fine crepitations all over the lung fields. Cardiovascular, neurological and abdominal examinations were unremarkable.

His arterial blood gas analysis showed a PaO_2_/FiO_2_ (P/F) ratio of 96. Oxygen therapy was instituted but his oxygenation failed to improve. The patient's trachea was then intubated for severe acute ARDS for oxygenation failure. Empirical therapy with meropenem, teicoplanin and oseltamivir was started. Enoxaparin 40mg subcutaneous once daily was started for deep vein thrombosis prophylaxis. The patient was sedated and proned for at least 16 hours a day based on the P/F ratio. Nasopharyngeal swab for influenza virus (H1N1) polymerase chain reaction was found to be positive. His initial arterial blood gas (ABG) analysis and other baseline investigations are as shown below (Table 1).

**Table 1:** Lab investigations at the time of admission.

Parameters	Value
WBC count	6900/cc
Differential count	N-77%, L-18%, M-5%
Platelets	196000/cc
Hb (PCV)	13.7g/dL (41.1%)
Total Bilirubin	0.3mg/dL
Direct Bilirubin	0.1mg/dl
SGPT	203IU/L
SGOT	248IU/L
ALP	147IU/L
Albumin	2.8g/dL
Urea	31mg/dL
Creatinine	0.9mg/dL
Serum sodium	144mEq/L
Seum potassium	2.9mEq/L
Cholride	108mEq.L
ABG	pH 7.47, PCO_2_ 34.6mm of Hg, HCO_3_ 24.9mmol/L, PaO_2_/FiO_2_= 96 (57.6/0.6)

**Figure 1:**
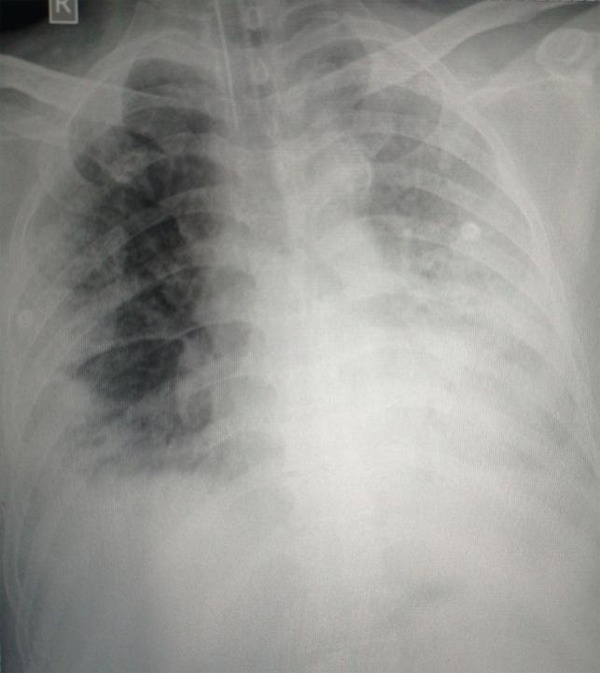
Bedside Portable Chest X-Ray Showing Bilateral Diffuse Lung Filed Infiltration.

Sputum, blood and urine bacterial cultures were sterile. His chest x-ray showed bilateral diffuse infiltration (Figure 1).

**Figure 2:**
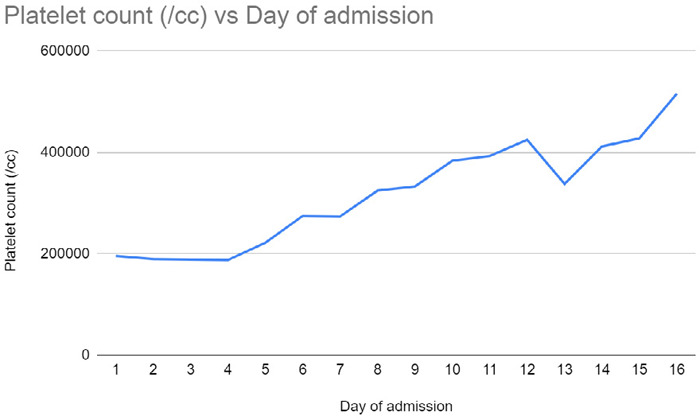
Daily ABG of the patient showing gradual improvement in P/F ratio.

Weaning from mechanical ventilation was gradually done. There was gradual improvement in the patient's P/F ratio as shown in (Figure 2).

What was notable was the platelet levels were within the normal range in the first few days and then rapidly increased by the beginning of the second week (Table 2).

**Table 2:** Gradual increase in the platelet count.

Day of admission	PaO_2_ (mm Hg)	FiO_2_	PaO_2_/FiO_2_ ratio
1	57.6	0.60	96.00
2	85.8	0.70	122.57
3	68.1	0.50	136.20
4	76.4	0.40	191.00
5	58.0	0.60	96.67
6	66.8	0.40	167.00
7	69.7	0.40	174.25
8	106.3	0.40	265.75
9	163.5	0.40	408.75

**Figure 3:**
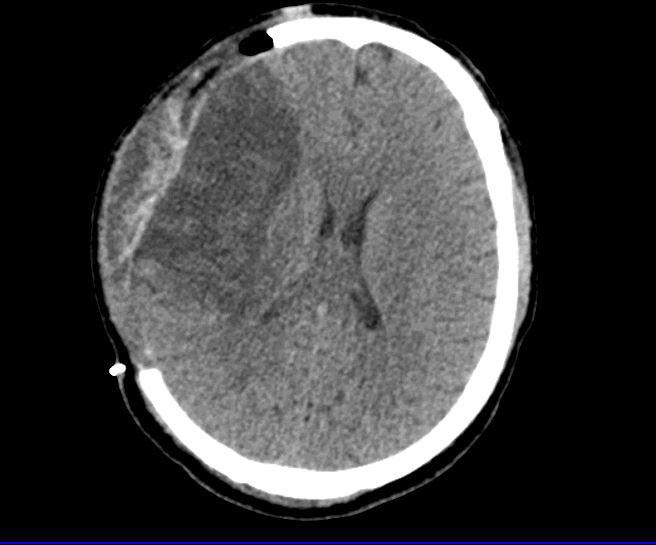
Post decompressive craniectomy CT head showing ischemic stroke in the right MCA territory with features of raised ICP.

Proning was stopped after day five following an improvement in the P/F ratio of more than 150 and the patient was given a spontaneous breathing trial (SBT) on day six after the FiO_2_ had decreased to 40% with a positive end expiratory pressure (PEEP) of 5cm of water. The SBT however failed due to a low Glasgow coma scale (GCS) of E2,Vt,M4. Left sided hemiplegia was noted. A computerized tomography (CT) head was obtained which showed a large ischemic stroke of the right middle cerebral artery (MCA) territory (Figure 3).

Hyperosmolar therapy with 3% sodium chloride was instituted. Neurosurgery consultation was obtained and urgent decompressive craniectomy was done. Post operatively the patient was kept intubated and weaning was started from the next day. There was gradual improvement in the GCS to E4,Vt,M5 by day 13 with stable oxygenation in 30-35% FiO2. He was successfully extubated on day 14. The GCS after extubating was E4,V2,M5. Physiotherapy was continued. Hyperosmolar therapy was continued for 10 days and then tapered in a few days. Physiotherapy was continued with gradual improvement in GCS to E4,V4,M6 and transferred to ward on day 7 of extubating and day 21 of admission. He was later discharged on day 29 of admission. He was put on a daily physiotherapy regime and a weekly follow up schedule for two weeks. Thereafter he had a follow up after 3 months for cranioplasty by which time his GCS was full with a slight weakness of the left half of the body and some slurring of vision.

## DISCUSSION

H1N1 influenza is a major cause of respiratory illnesses especially during outbreaks including epidemics and pandemics.^1^ However, these have been associated with other system involvements which are very less frequent and often under-reported. Neurological involvement has been associated with H1N1 infection though sparsely reported.^3,4^

Acute viremia itself can lead to altered sensorium in addition to pronounced hypoxemia in cases of severe pulmonary involvement. Various cytokines are released which cause a myriad of changes in the patient. Upregulation of various metalloproteinases, activation of coagulation cascade, endothelial injury, platelet aggregation and increased viscosity of the blood may be a few of the mechanisms responsible for ischemic events including MI and stroke.^5^ Severe ARDS has been associated with an estimated mortality of around 45%.^6^ The incidence of stroke associated with H1N1 infection is very rare with a reported risk of 0.6%.^4^

This case report is to underline the importance of the standard treatment of both ARDS and ischemic stroke in the overall survival of the patient. Both ARDS and MCA stroke are associated with high mortality and morbidity.^7,8^ The use of deep sedation and occasional paralysis during the management of ARDS with proning precluded the neurological assessment until the oxygenation had improved. Though a protocolized care of the patient is done with plans of daily sedation holidays and spontaneous awakening trials for all patients, there is a unique challenge in this category of patients because of the high oxygen needs and prone positioning. Until the ARDS improves, awakening the patient is very risky. Hence neurological assessment was not possible in the patient for the first five days.

The patient was also receiving DVT prophylaxis in the form of subcutaneous enoxaparin however this dose was not adequate to prevent stroke. Another thing that can be noted in our patient is that the platelet counts were within the normal range in the first few days and then rapidly increased by the beginning of the second week. Upregulation of cytokines and megakaryocytes have been implicated for this thrombocytosis. This can lead to a hypercoagulable status which could have been the cause for stroke.^5^ For this the patient was started and maintained on aspirin 75mg daily. This had however resolved to a normal platelet count by the time he was discharged.

Development of any new medical condition during the hospital especially in patients admitted in the ICU can be emotionally overwhelming to the patient and the family. This was such an event. Development of stroke during treatment of severe ARDS took a toll on the family, but the support and belief they endowed the medical team was very much appreciable. Timely management made it possible to successfully discharge the patient with acceptable neurological outcomes.
